# A prospective randomized study comparing amoxycillin/clavulanate (augmentin) with cefuroxime-metronidazole as prophylaxis in gynaecological surgery

**DOI:** 10.6026/973206300222578

**Published:** 2026-04-30

**Authors:** Shambhavi Soni, Pradeep Rathore, Ashis Tiwari

**Affiliations:** 1 Department of Obstetrics and Gynaecology, Government Medical College, Datia, Madhya Pradesh, India; 2 Department of General Surgery, Government Medical College, Datia, Madhya Pradesh, India; 3 Department of General Medicine, Government Medical College, Datia, Madhya Pradesh, India

**Keywords:** Gynecological surgery, antibiotic prophylaxis, amoxycillin-clavulanate, cefuroxime, metronidazole, surgical site infection (SSI)

## Abstract

Effective antibiotic prophylaxis is essential for preventing post-operative infections following gynecological surgery. This prospective randomized study conducted in a tertiary care hospital compared the efficacy of amoxicillin-clavulanate and cefuroxime-metronidazole as preoperative antibiotic regimens. Hence, two hundred women undergoing elective gynecological procedures were evenly divided into two groups: Group A received 1.2 g amoxicillin-clavulanate intravenously, while Group B received 1.5 g cefuroxime plus 500 mg metronidazole before surgical incision. Post-operative outcomes such as surgical site infection, febrile morbidity, urinary tract infections and duration of hospital stay were recorded and analyzed using SPSS, with significance set at p < 0.05. Thus, we show that both antibiotic combinations were equally effective in reducing post-operative infection rates and were well tolerated, supporting their safe use in gynecological surgery and the choice between regimens may depend on factors including hospital antibiotic policy, cost and local microbial resistance patterns.

## Background:

Hysterectomy, myomectomy, ovarian cystectomy and other pelvic operations are only a few examples of the many gynecological surgeries that are performed on a regular basis across the globe. Post-operative infections continue to be a major source of morbidity and extended hospital stays among women having these operations, despite the fact that surgical technology and aseptic measures have greatly decreased problems. Gynecological surgery patients are most often affected by surgical site infections (SSIs), urinary tract infections (UTIs) and febrile morbidity (Fever) after surgery [1]. Longer hospital stays; more antibiotic usage and more patient suffering are all consequences of post-operative infections. A number of factors, including the nature of the gynecological treatment, the patient's demographics and the effectiveness of post-operative infection prevention measures, influence the frequency of surgical site infections. Research on clean-contaminated gynecological operations has shown SSI incidences between 2% and 10% [2]. Antibiotic prophylaxis has greatly decreased post-operative infection problems and is now a common technique in contemporary surgical treatment. Giving antibiotics before microbial contamination occurs is known as prophylactic antibiotic medication and it helps reduce the risk of infection during and after surgical operations. To get the best preventative results, it's crucial to choose the right antibiotics at the right time [3]. Because they penetrate the vaginal system, which is home to a wide variety of microbes, gynecological operations are considered clean-contaminated procedures. Aerobic and anaerobic organisms including *Staphylococci*, *Streptococci*, *Bacteroides* species and *Escherichia coli* make up the vaginal flora. These microorganisms have the ability to induce infections after surgery if they are introduced into surgical incisions [4]. The perfect pre-operative antibiotic would be safe, effective against a wide variety of common infections, convenient to give and reach sufficient tissue concentrations during surgery without causing side effects. Prophylactic antibiotics such as beta-lactams and cephalosporins are often used during gynecological surgeries. These drugs are frequently mixed with others that kill anaerobic bacteria [5]. A beta-lactamase inhibitor and broad-spectrum penicillin make up the popular antibiotic combination amoxycillin-clavulanate, better known as Augmentin. By blocking the beta-lactamase enzymes generated by bacteria with resistance, clavulanic acid broadens amoxicillin's antibacterial range. When used together, these antibiotics are effective against a wide variety of surgical infection-causing microorganisms, both aerobic and anaerobic [6]. The use of cefuroxime in conjunction with metronidazole is another typical preventative strategy. The second-generation cephalosporin cefuroxime is effective against both aerobic and anaerobic bacteria, while metronidazole gives outstanding coverage against both types of germs. Procedures involving the female vaginal tract are well-suited to this combination because of the wide range of microbes it kills [7]. The selection of antibiotics used in gynecological surgeries varies greatly, even though antibiotic prophylaxis is commonly practiced. While some facilities favor amoxycillin-clavulanate and other single-agent therapies, others choose combination regimens such as cefuroxime and metronidazole. For the purpose of developing evidence-based recommendations and optimizing antibiotic use, comparative studies assessing the effectiveness of various regimens are crucial [8]. Additionally, sensible antibiotic selection is necessary for surgical prophylaxis due to the growing worry about antimicrobial resistance. The rise of antibiotic-resistant microorganisms, caused by either the excessive or improper use of these drugs, has the potential to raise healthcare expenses and make future treatments more difficult [9]. The risk of post-operative infections may be influenced by a number of variables, including antibiotic choice, time of administration, patient comorbidities, length of operation and adherence to aseptic procedures. To make sure there are enough antibiotics in the tissues when germs are present during surgery, it is best to give them 30-60 minutes before the incision is made [10]. Therefore, it is of interest to find out how well amoxycillin-clavulanate and cefuroxime-metronidazole work as preoperative antibiotics for women having gynecological surgeries and to determine the incidence of post-operative infections and any other clinical consequences linked to various antibiotic regimens.

## Materials and Methodology:

## Study design:

Located in the Obstetrics & Gynecology Department of a tertiary care teaching hospital, this research was planned as a prospective randomized comparative trial.

## Study duration:

Participant recruitment, surgery and post-operative follow-up all took place over the course of 18 months in the research.

## Study population:

Women having elective gynecological procedures such myomectomy, ovarian cystectomy, abdominal hysterectomy, or vaginal hysterectomy were part of the research.

## Sample size:

A total of 200 patients were included in the study. Participants were randomly divided into two groups:

[1] Group A (n = 100): Amoxycillin-clavulanate prophylaxis

[2] Group B (n = 100): Cefuroxime plus metronidazole prophylaxis

## Inclusion criteria:

[1] Women aged 18-60 years

[2] Patients undergoing elective gynecological surgery

[3] Patients who provided informed consent

[4] Patients with no evidence of active infection prior to surgery

## Exclusion criteria:

[1] Known allergy to beta-lactam antibiotics

[2] Patients with pre-existing infections

[3] Patients on antibiotic therapy before surgery

[4] Immunocompromised patients

[5] Emergency surgeries

## Randomization:

Participants were randomly allocated to either group using a computer-generated randomization method to ensure equal distribution.

## Antibiotic prophylaxis protocol:

[1] Group A: A 1.2 g intravenous dose of amoxycillin-clavulanate (Augmentin) was administered to patients thirty minutes prior to surgical incision.

[2] Group B: One half gram of cefuroxime and five hundred milligrams of metronidazole were given intravenously to the patients thirty minutes before to operation.

Additional post-operative doses were administered as per institutional protocol.

## Surgical procedure:

Skilled gynecological surgeons used rigorous aseptic technique throughout every operation. Every operation adhered to the accepted standards of surgical practice.

## Outcome measures:

## Primary outcome:

Incidence of surgical site infection (SSI) within 7 days after surgery.

## Secondary outcomes:

[1] Post-operative febrile morbidity

[2] Urinary tract infection

[3] Wound infection

[4] Duration of hospital stay

## Follow-up:

Patients were monitored daily during hospitalization and evaluated for signs of infection including:

[1] Fever

[2] Wound redness

[3] Purulent discharge

[4] Pain or swelling at the surgical site

Follow-up assessment was performed up to 7-10 days post-operatively.

## Data collection:

Clinical data were collected using a structured proforma including:

[1] Age

[2] Type of surgery

[3] Duration of surgery

[4] Post-operative complications

[5] Length of hospital stay

## Statistical analysis:

Data were entered and analyzed using (SPSS) software version 26.0. Descriptive statistics was used for mean, standard deviation, percentages and Chi-square test for categorical variables and Student's t-test for continuous variables. A p-value <0.05 was considered statistically significant.

## Results:

A total of 200 women undergoing elective gynecological surgery were included in the study. Participants were randomly allocated into two equal groups of 100 patients each. Group A: Amoxycillin-clavulanate (Augmentin), Group B: Cefuroxime + Metronidazole. The two groups were comparable with respect to demographic characteristics, type of surgery and duration of surgery. Both groups had comparable demographics. In Group A, the average age was around 41 years, whereas in Group B, it was 40.6 years. Abdominal hysterectomy (46-48% of all surgical procedures), vaginal hysterectomy (second most frequent) and ovarian cystectomy (third most common) were the next most prevalent. At the outset, there were no discernible differences between the two groups (p>0.05), suggesting that they were similar (Table 1 - see PDF). In both groups, the rate of post-operative infections was rather low. Among the patients who received Augmentin, 6% developed a surgical site infection; among those who received cefuroxime-metronidazole, 8% did so; and among those who had febrile morbidity, 7% and 9% did so, respectively. Statistical analysis revealed no significant differences (p>0.05) between the groups, suggesting that the two antibiotic regimens were equally efficient in avoiding post-operative infections (Table 2 - see PDF). The majority of patients (92% vs. 90%) had typical wound healing. Group B had a rate of delayed wound healing of 10% whereas Group A had an incidence of 8%. Although there was no statistically significant difference between Groups A and B, the average length of hospital stay was 4.6 days for Group A and 4.8 days for Group B (p>0.05) (Table 3 - see PDF). The efficacy of the Augmentin group in preventing infections was 94%, whereas the cefuroxime-metronidazole group achieved 92%. There was no statistically significant difference (p=0.59) between the groups, suggesting that the two antibiotic regimens were equally effective (Table 4 - see PDF).

## Discussion:

One of the leading causes of complications after gynecological surgery is surgical site infections. When given correctly before a surgical incision is made, prophylactic antibiotics considerably decrease the risk of post-operative infections. Preventative measures for gynecological procedures were studied in this prospective randomized trial using amoxycillin-clavulanate and cefuroxime-metronidazole. Although there was no statistically significant difference between the groups, 8% of patients in the cefuroxime-metronidazole group and 6% of patients in the Augmentin group had surgical site infections. Based on these results, it seems that both regimens are helpful in preventing infections after surgery. When comparing amoxycillin-clavulanate to cefuroxime and metronidazole for perioperative prophylaxis in gynecological surgery, a prospective randomized study conducted by van der Ban *et al.* found similar results. Based on their findings, the authors concluded that amoxycillin-clavulanate was just as effective as the combination treatment in terms of infection rates [11]. When comparing the efficacy of amoxycillin-clavulanate with that of cefuroxime and metronidazole, another randomized investigation found that both treatments were equally effective with comparable rates of febrile morbidity, UTIs and wound infections [12]. There was a low incidence of febrile morbidity (7% vs. 9%) in both groups, according to the current research. Previous clinical studies found fever morbidity rates of 5% to 10% in gynecological procedures with antibiotic prophylaxis, which is in line with our current results. Five percent of Augmentin patients and six percent of cefuroxime-metronidazole patients had urinary tract infections, respectively. Prior clinical investigations assessing prophylactic antibiotics in gynecological operations have also shown infection rates that are comparable [13]. Over 90% of patients had normal wound healing, which is another significant finding in this research. Wound healing delays were seen in fewer than 10% of instances, suggesting that the infection prevention measures were successful. Consistent with other reports of hospital stay lengths in patients undergoing simple gynecological operations given prophylactic antibiotics, the average length of time patients spent in the hospital during this research was four to five days. A single-dose prophylactic of amoxycillin-clavulanate has the potential to reduce antibiotic exposure and expenses during perioperative antibiotic therapy. To keep up proper coverage, however, the cefuroxime- metronidazole regimen often calls for repeated doses. In contrast, infections of the female vaginal tract often include both aerobic and anaerobic organisms; nonetheless, cefuroxime coupled with metronidazole provides broad-spectrum coverage against both types of bacteria [14]. This study's similar findings support the use of either regimen as a safe prophylactic measure in gynecological surgeries. Thus, antibiotic selection may be contingent upon variables such as institutional policies, regional patterns of microbial resistance, financial constraints and individual patient characteristics [15]. There were several restrictions on the current investigation. There was no evaluation of long-term infection outcomes as the follow-up period was restricted to the time immediately after surgery. Furthermore, not all infection cases had their microbiological culture data evaluated. The results provide important clinical evidence that both antibiotic regimes are successful in reducing post-operative infections in gynecological surgeries, despite these limitations.

## Conclusion:

Both amoxycillin-clavulanate and cefuroxime-metronidazole are equally effective prophylactic antibiotics in gynecological surgery, according to the current prospective randomized trial. Infections at the surgical site, febrile morbidity and UTIs were all rather rare with both treatments. The two medications have similar safety and effectiveness characteristics; however, amoxycillin-clavulanate may be easier to administer with a single dosage, while cefuroxime-metronidazole offers broader coverage against microbes. Antimicrobial stewardship principles, financial constraints and institutional rules should all be considered when selecting a prophylactic antibiotic.
